# Beyond diagnosis: the emerging role of fecal calprotectin in longitudinal management of pediatric juvenile polyps

**DOI:** 10.3389/fped.2026.1960219

**Published:** 2026-09-24

**Authors:** Toshihiko Kakiuchi

**Affiliations:** Department of Pediatrics, Faculty of Medicine, Saga University, Saga, Japan

**Keywords:** biomarker, children, fecal calprotectin, juvenile polyp, polypectomy, recurrence, surveillance

## Abstract

Fecal calprotectin (FCP) is an established noninvasive marker of intestinal inflammation, but its clinical relevance in children with juvenile polyps extends beyond the initial diagnostic evaluation. Available studies consistently show that FCP is elevated in most affected children and decreases markedly after complete polypectomy. Some data also suggest an association with polyp size, although this finding has not been reproduced in all cohorts. These observations suggest that FCP reflects lesion-associated inflammatory activity and the response to its removal. More limited longitudinal evidence suggests that re-elevation after initial normalization may accompany polyp recurrence. This mini-review examines the biological basis and clinical evidence for FCP in pediatric juvenile polyps across the disease course, from detection and assessment of lesion burden to post-polypectomy follow-up. Important uncertainties remain regarding the contribution of gastrointestinal bleeding, the mechanisms underlying markedly elevated values, and the accuracy and optimal timing of serial measurements. FCP should therefore be viewed as a promising adjunct rather than a substitute for clinical assessment and colonoscopy. Prospective studies are required to determine whether FCP trajectories can safely support longitudinal management and targeted post-polypectomy surveillance.

## 1 Introduction

Fecal calprotectin (FCP), a calcium- and zinc-binding protein derived predominantly from neutrophils, is an established noninvasive biomarker of intestinal inflammation. Stool FCP concentrations reflect neutrophil migration into the intestinal lumen and are widely used in pediatric practice to distinguish inflammatory bowel disease from functional gastrointestinal disorders, assess inflammatory activity, and help select children who require endoscopic evaluation (1, 2).

Juvenile polyps are the most common colorectal polyps in children and characteristically present with painless rectal bleeding. Colonoscopy provides both definitive diagnosis and endoscopic treatment. Complete examination of the colon is important because lesions may be multiple or located proximal to the rectosigmoid colon (3–6).

Accumulating evidence indicates that FCP is elevated in most children with juvenile polyps and declines substantially after complete polypectomy (7–10). A multicenter study found a strong positive correlation between FCP concentration and polyp size (9), whereas a subsequent larger single-center cohort confirmed higher FCP values in children with polyps but did not reproduce the size association (10). Thus, FCP may reflect lesion-associated inflammatory activity, although the determinants of its concentration remain incompletely defined.

Most published reports have evaluated FCP primarily as a diagnostic adjunct. However, the clinical meaning of the biomarker may extend across the patient trajectory: baseline elevation may support the decision to investigate, post-polypectomy normalization may indicate removal of the inflammatory source, and renewed elevation may raise concern for recurrence. Evidence for this longitudinal application remains preliminary, and FCP cannot currently replace clinical evaluation or colonoscopy.

This mini-review evaluates the biological basis and clinical utility of FCP in pediatric juvenile polyps, with particular emphasis on its evolution from a diagnostic marker toward a potential biomarker of treatment response and post-polypectomy surveillance. We also identify the evidence gaps that must be addressed before serial FCP testing can be incorporated into routine longitudinal management. The current clinical evidence and implications are summarized in Table 1.

**Table 1:** Clinical evidence and implications of fecal calprotectin in pediatric juvenile polyps.

Clinical question	Current evidence	Clinical implication
Is FCP elevated?	FCP is higher in children with juvenile polyps than in children with normal colonoscopic findings; normal values appear uncommon in affected cohorts (7–10).	Consider juvenile polyps in children with unexplained FCP elevation, especially when painless rectal bleeding is present. FCP is not diagnostic in isolation.
Does FCP reflect lesion burden?	One multicenter study found a strong correlation with polyp size (9), but a larger subsequent cohort did not reproduce associations with size, number, type, or location (10).	FCP may reflect lesion-associated inflammatory activity, but it should not be used to estimate polyp size or number.
Does FCP decrease after polypectomy?	FCP decreases substantially and often normalizes after complete resection (7–9, 12–14).	A post-polypectomy measurement may provide noninvasive confirmation of treatment response when interpreted with clinical and endoscopic findings.
Can FCP detect recurrence?	Recurrence occurs after removal (16, 17), and a longitudinal case showed normalization, re-elevation with recurrence, and renormalization after repeat resection (18).	Serial monitoring is promising but remains investigational and cannot replace symptom assessment or colonoscopy.

FCP, fecal calprotectin.

## 2 Biological basis of fecal calprotectin elevation

Calprotectin is released mainly from activated neutrophils during mucosal inflammation and constitutes a large proportion of their soluble cytosolic protein content. Its resistance to degradation and abundance in neutrophils make stool concentration a useful surrogate for neutrophil recruitment to the gastrointestinal mucosa. FCP is therefore an inflammation marker rather than a disease-specific marker (1, 11).

Histologically, juvenile polyps contain cystically dilated glands within an edematous and inflamed lamina propria. Surface erosion, ulceration, granulation tissue, and neutrophilic infiltration may be prominent (3, 4, 6). These features provide a biologically plausible source of FCP. The consistent reduction in FCP after complete polypectomy further supports the lesion itself as the principal source of the elevation (7–9, 12–14).

Nevertheless, FCP values may appear disproportionate to the limited surface area of a solitary lesion. Concentrations exceeding 1,000 µg/g have been documented in children with solitary or large juvenile polyps (9, 12–14). Several non-mutually exclusive mechanisms may account for this observation. A small lesion may contain intense neutrophilic inflammation; surface ulceration may permit continuous release of inflammatory material into the lumen; and lesion size may incompletely represent the biologically active surface. These explanations remain hypotheses because no study has directly correlated FCP with neutrophil density, ulcerated surface area, or a validated histological inflammation score in juvenile polyps.

Gastrointestinal bleeding is another potential contributor. Experimental ingestion of blood by healthy adults can raise FCP, particularly after a large blood load, demonstrating that blood in the gastrointestinal tract may influence the measurement (15). However, that model involved upper gastrointestinal blood ingestion and cannot be directly extrapolated to the usually smaller-volume lower gastrointestinal bleeding caused by juvenile polyps. Existing pediatric polyp studies were not designed to separate the effect of bleeding from local mucosal inflammation (7–10, 12–14). The marked fall after polypectomy favors removal of an inflammatory lesion as the dominant explanation, but the relative contributions of bleeding and tissue inflammation remain unresolved.

Clarifying this biological basis is clinically relevant. If FCP is to be interpreted longitudinally, clinicians must understand whether changes primarily reflect lesion presence, inflammatory intensity, bleeding, or a combination of these factors.

## 3 Clinical utility across the disease course

### 3.1 Diagnostic evaluation

Case reports first drew attention to marked FCP elevation in children with juvenile polyps and normalization after removal (12–14). Subsequent observational studies demonstrated higher FCP concentrations in children with juvenile polyps than in children with normal colonoscopic findings (7–10). In the largest cohort reported to date, only a small minority of children with polyps had FCP below the laboratory reference limit, and receiver operating characteristic analysis showed moderate diagnostic discrimination (10).

FCP is nevertheless nonspecific. Elevated values are also encountered in inflammatory bowel disease, infection, medication-associated enteropathy, and other inflammatory conditions (1, 2, 11). FCP should therefore not be used to diagnose a juvenile polyp in isolation. Its practical value is to broaden the differential diagnosis of unexplained elevation and to strengthen the indication for colonoscopy in a child with compatible symptoms, particularly painless rectal bleeding. A normal result may reduce the likelihood of a polyp, but currently available data do not justify using FCP alone to exclude the diagnosis.

### 3.2 Lesion-associated inflammatory burden

The possibility that FCP reflects more than lesion presence is clinically attractive. In a multicenter cohort of 33 children, polyp size was the only variable independently associated with FCP, and the correlation was strong (9). Reports of large juvenile polyps with markedly elevated values are consistent with this observation (12–14).

However, the association should not be regarded as established. The larger 2025 cohort did not identify correlations with polyp size, number, histological type, or location (10). Differences in study design, assay timing, lesion characteristics, bleeding, and sample size may account for the discordant findings. At present, FCP may indicate lesion-associated inflammatory burden, but it cannot reliably estimate polyp size or number in an individual patient. Direct correlation with endoscopic ulceration and histopathological inflammation is a priority for future research.

### 3.3 Response after polypectomy

A substantial decline, often to the normal range, after complete polypectomy is one of the most reproducible findings in the literature (7–9, 12–14). In the multicenter study by Kim et al., median FCP decreased from 445.2 to 26.5 mg/kg among children with paired measurements (9). This consistent biological response provides stronger evidence than the cross-sectional association with lesion size.

Post-polypectomy normalization may therefore function as a noninvasive indicator that the inflammatory source has been removed. Persistent elevation should not automatically be interpreted as residual polyp tissue, because intercurrent infection, inflammatory bowel disease, medication exposure, and incomplete mucosal healing are alternative explanations. Nevertheless, when interpreted alongside symptoms and the completeness of colonoscopy, a post-treatment measurement may provide clinically useful confirmation of response.

### 3.4 Recurrence and post-polypectomy surveillance

Recurrent or metachronous juvenile polyps are clinically relevant. Pediatric series have documented recurrence after apparently complete removal in children with both solitary and multiple polyps (16, 17). However, these studies predated routine FCP use and did not evaluate a biomarker-guided surveillance strategy.

Current follow-up after polypectomy is based primarily on clinical assessment and, in selected patients, repeat colonoscopy. After complete removal of a single juvenile polyp in a child without suspicious histological features or a relevant family history, routine repeat colonoscopy is generally not required, and reassessment is prompted by recurrent symptoms, particularly rectal bleeding (19). In contrast, children with multiple juvenile polyps require closer endoscopic follow-up because of the greater risk of recurrence and the possibility of juvenile polyposis syndrome (16, 19). Thus, any potential role for serial FCP should be considered within, rather than as a replacement for, these established clinical and endoscopic approaches.

Longitudinal evidence for FCP is currently limited to case-level observations. In our previously reported patient, FCP normalized after initial polypectomy, rose again when a recurrent solitary juvenile polyp was detected, and returned to the normal range after repeat removal (18). This temporal pattern supports the concept that the trajectory of FCP—not merely a single measurement—may contain clinically useful information.

These case-level observations should be regarded as hypothesis-generating rather than evidence of an established surveillance strategy. FCP-based surveillance therefore remains investigational and cannot currently be recommended for routine clinical practice. No validated threshold or change from baseline has been established for recurrence detection, and its sensitivity, specificity, and optimal timing after polypectomy remain unknown.

## 4 Unresolved questions and research priorities

### 4.1 What determines marked elevation?

The highest FCP values cannot currently be explained by polyp size alone. Studies should integrate pre-polypectomy FCP with lesion diameter, location, number, degree of surface erosion or active bleeding, and quantitative histological assessment. Such analyses could determine whether the biomarker reflects inflammatory cell density, exposed inflammatory surface, bleeding, or an interaction among these factors. Standardized processing of resected specimens and blinded pathology scoring would be essential.

### 4.2 How much does bleeding contribute?

The effect of blood is a potential confounder when FCP is interpreted in a child presenting with hematochezia. Experimental evidence confirms that a large gastrointestinal blood load can elevate FCP (15), but the magnitude and duration of this effect in lower gastrointestinal bleeding are unknown. Future studies should record bleeding severity and contemporaneous fecal hemoglobin, allowing the independent associations of bleeding and lesion inflammation with FCP to be evaluated.

### 4.3 Can a clinically useful surveillance strategy be defined?

A surveillance strategy requires more than evidence that FCP decreases after treatment. Prospective cohorts should obtain a standardized post-polypectomy baseline and then measure FCP at predefined intervals, with recurrence confirmed by colonoscopy. Multicenter collaboration will probably be required because juvenile polyps are relatively uncommon and recurrence is infrequent. Analyses should evaluate absolute thresholds, change from the individual baseline, and repeated upward trends, and should determine the sensitivity, specificity, positive and negative predictive values, and optimal timing of FCP measurements for recurrence detection. Because FCP is nonspecific, a clinically useful algorithm will probably combine biomarker trajectory with recurrent bleeding, anemia, and other symptoms rather than rely on a single universal cutoff.

The principal clinical outcome should be whether such an approach reduces unnecessary colonoscopy without delaying the diagnosis of recurrence. Diagnostic accuracy, patient acceptability, cost, and the consequences of false-positive and false-negative results should all be assessed before implementation.

## 5 Future perspectives

Three priorities emerge from the current evidence. First, clinicopathological studies should define the biological determinants of FCP elevation by linking stool concentrations with endoscopic and quantitative histological findings. Second, prospective longitudinal studies should characterize the expected FCP trajectory after complete polypectomy and during recurrence. Third, clinically actionable algorithms should be developed and validated, specifying when a post-polypectomy result is reassuring and when persistent or renewed elevation should prompt further evaluation.

These studies should distinguish sporadic juvenile polyps from juvenile polyposis syndromes and should account for assay variability, age, infection, medication exposure, and other causes of intestinal inflammation. Until such evidence is available, FCP is best used as an adjunct that complements—not replaces—clinical assessment and endoscopy.

## 6 Conclusion

Fecal calprotectin is consistently elevated in many children with juvenile polyps and declines after complete polypectomy, supporting its clinical value beyond the initial diagnostic evaluation. Evidence for an association with lesion burden is suggestive but inconsistent, and evidence for recurrence detection remains preliminary. The most promising future application is longitudinal: establishing whether normalization confirms treatment response and whether renewed elevation can identify children who warrant targeted reassessment. Rather than serving solely as a diagnostic biomarker, FCP may ultimately become a useful component of post-polypectomy management, but prospective validation will determine whether FCP can evolve from a diagnostic adjunct into a practical biomarker for longitudinal management of pediatric juvenile polyps.

## References

[1] Koninckx CR Donat E Benninga MA Broekaert IJ Gottrand F Kolho K-L. The use of fecal calprotectin testing in paediatric disorders: a position paper of the European Society for Paediatric Gastroenterology and Nutrition Gastroenterology Committee. J Pediatr Gastroenterol Nutr. (2021) 72:617–40. 10.1097/MPG.0000000000003046 33716293

[2] Orfei M Gasparetto M Hensel KO Zellweger F Heuschkel RB Zilbauer M. Guidance on the interpretation of faecal calprotectin levels in children. PLoS One. (2021) 16:e0246091. 10.1371/journal.pone.0246091 33571226 PMC7877663

[3] Thakkar K Fishman DS Gilger MA. Colorectal polyps in childhood. Curr Opin Pediatr. (2012) 24:632–7. 10.1097/MOP.0b013e328357419f 22890064

[4] Kay M Eng K Wyllie R. Colonic polyps and polyposis syndromes in pediatric patients. Curr Opin Pediatr. (2015) 27:634–41. 10.1097/MOP.0000000000000265 26208235

[5] Lee BG Shin SH Lee YA Wi JH Lee YJ Park JH. Juvenile polyp and colonoscopic polypectomy in childhood. Pediatr Gastroenterol Hepatol Nutr. (2012) 15:250–5. 10.5223/pghn.2012.15.4.250 24010095 PMC3746052

[6] Vermeulen D Van Winckel M Vande Velde S De Bruyne R Van Biervliet S De Moerloose B. Not all pediatric intestinal polyps are alike. Acta Gastroenterol Belg. (2020) 83:393–7. 33094585

[7] Olafsdottir I Nemeth A Lörinc E Toth E Agardh D. Value of fecal calprotectin as a biomarker for juvenile polyps in children investigated with colonoscopy. J Pediatr Gastroenterol Nutr. (2016) 62:43–6. 10.1097/MPG.0000000000000893 26147630

[8] Das SR Karim ASMB RukonUzzaman M Mazumder MW Alam R Benzamin M. Juvenile polyps in Bangladeshi children and their association with fecal calprotectin as a biomarker. Pediatr Gastroenterol Hepatol Nutr. (2022) 25:52–60. 10.5223/pghn.2022.25.1.52 35087733 PMC8762599

[9] Kim YB Kim JY Choi S Lee YM Choi SY Kim SC. Fecal calprotectin levels significantly correlate with polyp size in children and adolescents with juvenile colorectal polyps. Pediatr Gastroenterol Hepatol Nutr. (2023) 26:34–42. 10.5223/pghn.2023.26.1.34 36816439 PMC9911176

[10] Kolobarić A Mažuranić IT Ruška P Pavić AM Mišak Z Hojsak I. Can fecal calprotectin guide the need for colonoscopy in children with suspected polyps? Eur J Pediatr. (2025) 184:662. 10.1007/s00431-025-06503-z 41055803

[11] Ayling RM Kok K. Fecal calprotectin. Adv Clin Chem. (2018) 87:161–90. 10.1016/bs.acc.2018.07.005 30342711

[12] Khan F Mani H Chao C Hourigan S. Fecal calprotectin as a future screening tool for large juvenile polyps. Glob Pediatr Health. (2015) 2:2333794X15623716. 10.1177/2333794X15623716 27335988 PMC4784632

[13] Pauley-Hunter RJ Kunnath S Wolff K Vanderhoof JA. Fecal calprotectin and pediatric juvenile polyps. J Pediatr Gastroenterol Nutr. (2015) 60(4):e30–1. 10.1097/MPG.0000000000000173 24048165

[14] Kim KY Kim JS. Successful detection and removal of predictable juvenile polyp: a case report. Ann Coloproctol. (2023) 39(5):435–8. 10.3393/ac.2021.00311.0044 34284556 PMC10626337

[15] Vavricka SR Heinrich H Buetikofer S Breitenmoser F Burri E Schneider-Yin X. The vampire study: significant elevation of faecal calprotectin in healthy volunteers after 300mL blood ingestion mimicking upper gastrointestinal bleeding. United European Gastroenterol J. (2018) 6:1007–14. 10.1177/2050640618774416 30228888 PMC6137587

[16] Fox VL Perros S Jiang H Goldsmith JD. Juvenile polyps: recurrence in patients with multiple and solitary polyps. Clin Gastroenterol Hepatol. (2010) 8:795–9. 10.1016/j.cgh.2010.05.010 20580940

[17] Pillai RB Tolia V. Colonic polyps in children: frequently multiple and recurrent. Clin Pediatr (Phila). (1998) 37:253–7. 10.1177/000992289803700406 9564575

[18] Kudoh M Kakiuchi T Yoshiura M Esaki M Matsuo M. Fecal calprotectin measurement to detect recurrence of solitary juvenile polyps: a case report. Medicine (Baltimore). (2023) 102:e35448. 10.1097/MD.0000000000035448 37904372 PMC10615447

[19] Cohen S Hyer W Mas E Auth M Attard TM Spalinger J. Management of juvenile polyposis syndrome in children and adolescents: a position paper from the ESPGHAN polyposis working group. J Pediatr Gastroenterol Nutr. (2019) 68:453–62. 10.1097/MPG.0000000000002246 30585890

